# Effectiveness of escape room–based learning for patient safety education in nursing students

**DOI:** 10.1186/s12909-026-09046-5

**Published:** 2026-03-23

**Authors:** Inangil Demet, Semiz Demet, Turkmen Kübra, Kokkiz Rukiye

**Affiliations:** 1 Fundamental of Nursing Department, Hamidiye Faculty of Nursing, Sağlık Bilimleri University, 38 Tıbbiye Street, Uskudar, Istanbul, 34668 Turkey; 2 Lecturer Department of First and Emergency Aid, Vocational School of Health Services, Fenerbahçe University, Ataturk Dıstrıct, Atasehir Boulevard, Ataşehir, Istanbul, 34758 Turkey; 3 Nursing Department, Hamidiye Sağlık Bilimleri Institute, Sağlık Bilimleri University, Uskudar, Istanbul, 34668 Turkey

**Keywords:** Patient safety, Escape room, Nursing education, Nursing students, Teaching methods.

## Abstract

**Background:**

Ensuring patient safety is a cornerstone of nursing practice, and its foundation is established during nursing education. The study evaluates the effectiveness of escape room–based learning designed in accordance with the International Patient Safety Goals on nursing students’ self-efficacy about patient safety.

**Methods:**

The study was conducted with senior nursing students from February to May 2024. Data were collected using the Student Information Form, Patient Safety Self-Efficacy Questionnaire, and Satisfaction with Training Methods Scale. The escape room was structured in three phases—prebriefing, simulation, and debriefing—following INACSL standards. Clinical trial number: NCT07179367.

**Results:**

The average age of the participants was 23.5, and 43.5% had previously completed an elective course on patient safety. Analyses revealed that participants spent the most time, averaging 2.59 min, in the room dedicated to information questions. The average self-efficacy score before the intervention was 61.26, which increased to 71.32 after the intervention, and participants reported a high level of satisfaction.

**Conclusion:**

This study’s findings indicate that patient safety-themed escape room training significantly enhances students’ self-efficacy in patient safety, as well as their overall satisfaction and motivation.

**Supplementary Information:**

The online version contains supplementary material available at 10.1186/s12909-026-09046-5.

## Background

Everyone has the fundamental right to live in a healthy and safe environment [1]. However, safety issues that arise within healthcare settings can compromise this right, posing significant risks to patient well-being. The US National Patient Safety Foundation (NPSF) underscores the critical nature of patient safety, defining it as “the prevention of errors related to healthcare and the reduction of patient harm caused by such errors” [2]. Given nurses’ sustained and direct contact with patients, they are central to preventing harm and promoting patient safety across the continuum of care. Accordingly, nursing curricula should intentionally embed patient safety culture through student-centered, practice-oriented educational strategies before students enter clinical settings [3, 4]. Joint Commission International (JCI) (2011) has established “International Patient Safety Goals,” which include correct patient identification, enhanced communication, improved safety of high-risk medications, correct-site and correct-procedure surgery, reduction of healthcare-associated infections, and minimization of patient harm from falls. To achieve these goals, JCI advocates for the adoption of active teaching methods that holistically address the cognitive, affective, and psychomotor dimensions of patient safety in health education [5].

Active teaching methods that directly engage students and cater to diverse learning styles are increasingly recognized as essential in nursing education [6]. Among these methods, gamification—transforming educational content into interactive and engaging experiences—has gained prominence. Gamification, such as the “Escape Room” approach, enhances learning by transforming passive students into active participants, thereby making the educational process more dynamic and effective [7]. In an escape room scenario, teams of 2–10 members work together to solve puzzles and complete tasks, progressing through multiple rooms with time constraints and optional hints for time management [8]. This method has been shown to improve students’ cooperation, communication, critical thinking, and active learning, while also enhancing their motivation, knowledge acquisition, and team-building skills [9].

The escape room approach is increasingly being utilized across various healthcare disciplines, including nursing, medicine, pharmacy, physiotherapy, and pharmacology [10]. Studies on the use of this method in nursing education show that simulation-based escape rooms are effective in identifying patient safety risks, increasing students’ self-confidence, and providing interdisciplinary learning opportunities by emphasizing that safety culture is a shared responsibility [11, 12]. Moreover, studies have shown that nursing students not only increase their awareness of patient safety through active participation in escape rooms but also find the experience enjoyable and motivating. For instance, Dacanay et al. reported that an escape room themed around sepsis care enhanced students’ ability to retain and apply critical thinking skills, leading to positive outcomes in both knowledge retention and skill development [13]. Similarly, Gracia et al. found that nursing students had fun and effectively acquired professional competencies while playing an escape room game during a community health course, validating the use of gamification as a teaching strategy [14].

This study focuses on patient safety in nursing education and aims to enhance students’ patient safety competencies through active teaching methods. The intervention is grounded in gamification, escape room pedagogy, and experiential learning theory. Specifically, the study evaluates the impact of an escape room activity designed in accordance with the International Patient Safety Goals on nursing students’ self-efficacy about patient safety. Aligned with experiential learning theory, this approach offers a holistic learning process that extends beyond technical skill acquisition and incorporates cognitive, psychological, social, and emotional dimensions of learning [9, 12].

## Method

### Design

**Fig. 1:**
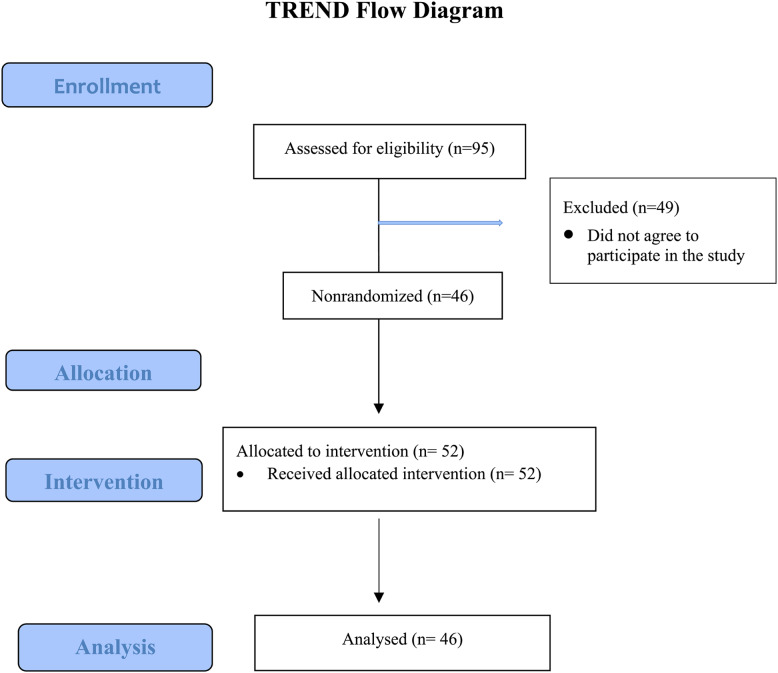
Allocation of subjects according to the TREND Flow diagram

The study was conducted in a quasi-experimental type with a one-group pretest-posttest design. The study complied with the TREND Checklist guidelines, and the research flow diagram created by the researchers was based on TREND (Fig. 1). Figure 1 shows the distribution of subjects according to the TREND Flowchart.

### Sample

The study population included senior students at XX Faculty of Nursing, University of XX, between February 2024 and May 2024 (*N* = 95). Students who voluntarily agreed to participate were included in the sample without sample size calculations, as the goal was to reach the entire population. The inclusion criteria were (a) being 18 years of age or older, (b) being a fourth-year nursing student, (c) completing the data collection forms in full, and (d) volunteering to participate in the study. Being in the escape room for more than 6 min in each room was set as an exclusion criterion. The study was completed with 46 participants (Fig. 1). Figure 1 shows the distribution of subjects according to the TREND Flowchart. A post hoc power analysis conducted after data collection showed that the study had a statistical power of 0.90 at a significance level of 0.05 and an effect size of 0.5.

### Hypothesis

- H_1_^1^ Escape room teaching method increases students’ self-efficacy in patient safety practices in nursing students.
- H_1_^2^ Escape room teaching method increases nursing students’ satisfaction with the patient safety teaching method.

### Data collection tools

As data collection tools in this research student Information Form, Patient Safety Self-Efficacy Questionnaire and Satisfaction with Training Methods were used.

#### Student information form

The form, developed by the researchers in line with the literature, consists of questions containing demographic data to determine the participants’ age, gender, type of school graduated from, and whether they have taken elective courses on patient safety [15, 16].

#### Patient safety self-efficacy questionnaire

The structural content of the patient safety self-efficacy questionnaire created by the researchers is referenced from Eskici et al. and consists of 15 items [17]. The corrected item-total correlations of the items range from 0.748 to 0.918. This indicates that the items are highly consistent overall and have very good discriminatory power. In the factor analysis conducted to evaluate construct validity, the KMO value was found to be 0.916 and the Bartlett test was statistically significant (χ²=961.332 *p* <.001). Cronbach’s Alpha value was found to be 0.977.

It includes a degree of competence between 0 and 75 (0 = insufficient, 5 = sufficient) for each goal. Fifteen headings were determined by matching the keywords in the ‘International Patient Safety Goals’ set by JCI (Table 1). These headings were finalised by consulting five experts in the field. A minimum score of 0 and a maximum score of 75 can be obtained from the questionnaire. High scores will indicate high self-efficacy for patient safety. Low scores would indicate low self-efficacy for patient safety. This questionnaire was selected as it evaluates self-efficacy related to patient safety by assessing individuals perceived capability to perform essential safety behaviors. Given that its items correspond to core patient safety competencies such as safe communication, medication administration, infection control, and risk prevention, it was considered appropriate for evaluating outcomes of an escape room–based patient safety intervention.

**Table 1:** Patient safety self-efficacy headings

1. I can establish safe communication that can ensure patient safety.
2. I can cooperate with other disciplines for patient safety.
3. I can use reporting systems for patient safety.
4. I can make safe patient transfers.
5. I can determine the safe site when practicing giving injections to the patient.
6. I can calculate the safe dose range in drug applications.
7. I can take the necessary security measures to verify patient identity.
8. I can use colour codes (red-white-blue-pink) in the hospital.
9. I can ensure patient privacy safely.
10. I can take precautions for falls in patient safety.
11. I can recognise the safe connections of medical materials given to the patient.
12. I can recognise applications that may infect the patient.
13. I can ensure the safety of medical supplies given to the patient in terms of infection.
14. I can recognise situations that may threaten patient safety related to the patient’s environment.
15. I can take precautions for pressure injuries in patient safety.

#### Satisfaction with training methods scale

The scale was developed by Gurpinar (Cronbach’s alpha: 0.84) and used in their studies. In this study, the Cronbach’s alpha value of the scale is 0.768. It contains 16 items, with each proposition scored on a 5-point Likert scale (1 = strongly disagree, 2 = disagree, 3 = undecided, 4 = agree, 5 = strongly agree). High average scores indicate that students’ satisfaction with the education method is high. Low average scores indicate that students’ satisfaction with the education method is low. Students can rate their satisfaction with the training methods with a minimum of 16 and a maximum of 80 points [18].

### Interventions

All participants who volunteered for the study were thoroughly briefed on the duration, flow, and expectations of the interventions. Both verbal and written consent were obtained from each participant. The date and time for the escape room activity were scheduled, and students were notified accordingly. Participants were required to assemble into teams of four to six members and report to the Nursing Simulation Laboratory within the designated time frame, as this team size was selected to promote effective communication, collaboration, and active participation, which are core components of patient safety practices.

The Patient Safety Self-Efficacy Questionnaire was administered as a pre-test prior to the commencement of the escape room activity. The design and implementation of the escape room adhered to the ‘Best Practice Standards’ established by the International Nursing Association for Clinical Simulation and Learning (INACSL) Standards Committee and the INACSL Board of Directors [19].

The intervention was structured in three phases: prebriefing, simulation training (Escape Room), and debriefing. Following the debriefing session, participants completed the Patient Safety Self-Efficacy Questionnaire and the Satisfaction with Training Methods Questionnaire.

### Escape room design

The research was conducted by three academics and one intensive care nurse. The researchers were involved in and actively participated in all stages of the research. The patient safety–themed escape room scenario was systematically designed based on the International Patient Safety Goals defined by the Joint Commission International (JCI). Fifteen patient safety domains were identified and matched with the items of the Patient Safety Self-Efficacy Questionnaire (Table 1). Each room and task was explicitly mapped to these predefined domains to ensure content alignment and standardization across participants. The scenario structure, task difficulty, time limits, and expected responses were identical for all student groups. Content validity and scenario coherence were evaluated by five experts in nursing education and patient safety, and the final version of the escape room was refined based on their feedback.

The escape room was created in three stages: prebriefing, simulation and debriefing, under INACSL standards [19].

### A- Prebriefing

Prior to the commencement of the escape room simulation, students underwent a comprehensive orientation session. This session included:

- Introduction to the simulation environment and management procedures within the simulation laboratory.
- Provision of foundational information regarding patient safety principles that would be integrated into the escape room experience.
- Explanation of the objectives and expected outcomes for the students (Table 2).
- Obtaining consent for video and photo documentation of the simulation.
- Emphasis on the importance of confidentiality concerning the escape room’s design and content to prevent information leakage.
- Detailed description of the escape room setup: the laboratory was divided into three distinct rooms.

**Table 2:** The objectives and expected outcomes for the students according to jci patient safety goal

JCI Patient Safety Goal	Escape Room Room	Escape Room Practices	Expected Learning Outcomes	Simulation Practice Objectives
Goal 1: Identify patients correctly	Second Room (Mother–Infant Room)	Distinguishing mother–infant patient wristbands	Recognizing correct patient identification.	Raising awareness of identification verification practices
Goal 1: Identify patients correctly	Second Room (Mother– Infant Room)	Use of hospital color codes (red, white, blue, pink)	Accurate identification of the patient and risks.	Identification of risks related to patient safety and taking appropriate precautions
Goal 2: Improve effective communication	First Room (Patient Safety – Information Notes)	Identification of practices ensuring safe communication	Recognizing the importance of safe and effective communication.	Promotion of teamwork and effective communication
Goal 2: Improve effective communication	First Room (Patient Safety – Information Notes)	Identification of practices supporting interdisciplinary collaboration	Recognizing internal team communication	Promotion of teamwork and effective communication
Goal 2: Improve effective communication	First Room (Patient Safety – Information Notes)	Identification of patient safety reporting systems	Communication awareness through reporting	Promotion of teamwork and effective communication
Goal 3: Improve the safety of high-risk medications	Second Room (Mother– Infant Room)	Calculation of safe medication dose range	Awareness regarding drug safety.	Reinforcement of safe medication practices and raising awareness
Goal 4: Identify and assess risks	First Room (Patient Safety – Information Notes)	Demonstration of safe injection site from the ventrogluteal region	Identifying secure startup practices	Introduction of basic patient safety principles to students and adoption of safe practices
Goal 4: Identify and assess risks	Third Room (Adult Intensive Care Unit)	Identification of cables and connections passing under the patient	Recognizing the risks of pressure injuries.	Identification of patient safety risks in different clinical settings
Goal 4: Identify and assess risks	Third Room (Adult Intensive Care Unit)	Recognition of sharp objects on the patient’s bed	Identifying environmental safety risks.	Introduction of basic patient safety principles and environmental risks to students
Goal 4: Identify and assess risks	Second Room (Mother– Infant Room)	Practices related to ensuring maternal privacy	Raising awareness about safe and ethical patient care.	Ensuring patient privacy and raising awareness
Goal 5: Reduce the risk of health care–associated infections	Third Room (Adult Intensive Care Unit)	Recognition of dirty and bloody peripheral catheter dressing	Recognizing infection risks	Identification of patient safety risks related to infection control
Goal 5: Reduce the risk of health care–associated infections	Third Room (Adult Intensive Care Unit)	Identification of oxygen mask in contact with the floor	Recognizing the risks associated with infection control.	Identification of patient safety risks related to infection control
Goal 6: Reduce the risk of patient harm resulting from falls	Second Room (Mother– Infant Room)	Identification of safety risks related to open incubator edges	Recognizing the risks of falls and injuries.	Identification of patient safety risks in different clinical settings
Goal 6: Reduce the risk of patient harm resulting from falls	First Room (Patient Safety – Information Notes)	Identification of practices for safe patient transfer	Recognizing physical harm and fall risks.	Identification of patient safety risks in different clinical settings

The first room featured four knowledge-based questions and one skill-based question, the second room addressed medication dosage calculations and patient safety issues related to mother-infant care, and the third room focused on patient safety concerns within an intensive care setting. Students were informed that they would have a maximum of six minutes in each room. Correct responses to the questions were assigned letters, and the completion of five correct answers would form the word “T-A-M-A-M” (Turkish for “OKAY”), which served as the key to proceed to the next room. Successful completion of all three rooms would be followed by evaluation based on the time spent in each room. A facilitator was available to provide hints if students exceeded the time limit of five minutes per room.

### B- Simulation - escape room

#### First room (Patient safety - knowledge notes)

Upon entering the room, the timer was activated. Students were tasked with answering five patient safety-related questions:

1. List three practices that ensure safe communication in patient safety.
2. Identify three practices for effective inter-disciplinary collaboration in patient safety.
3. Name three reporting systems used for patient safety.
4. Describe three practices that ensure safe patient transfer.
5. Demonstrate the safe injection site in the gluteal area (ventrogluteal area) using a skill model.

#### Second room (Mother-infant room)

In this room, students were required to perform a medication dosage calculation relevant to patient safety and identify four patient safety threats:

1. Calculation of a safe drug dose range.
2. Identification of different mother-infant patient wristbands.
3. Utilization of color codes (red, white, blue, pink) in the hospital setting.
4. Ensuring maternal privacy.
5. Addressing safety concerns with open incubator edges.

#### Third room (Adult intensive care unit)

- Students were asked to identify five practices that pose threats to patient safety:
  1. Incorrect connection of medical equipment (e.g., intravenous treatment administered through a nasogastric catheter).
  2. Presence of bloody and dirty peripheral catheter dressing.
  3. Oxygen mask tip exposed and in contact with the floor.
  4. Sharp objects present on the patient’s bed.
  5. Patient cables and connections passing under the patient, risking pressure injuries.

### C- Debriefing

- The debriefing phase utilized the Promoting Excellence and Reflective Learning in Simulation (PEARLS) method [20]. This method encompasses four stages: reaction, definition, analysis, and summarization.
- The information phase was conducted by three academics with intensive care certification. The interview was conducted verbally.
- During this phase, students engaged in reflective discussion about their experiences, including their practices, decision-making processes, and the outcomes of the simulation exercise. The aim was to facilitate a thorough understanding and learning from their simulation experience.

### Legal and ethical aspects of the research

Permission was obtained from the University of XX, XX Scientific Research Ethics Committee, and the institution where the research was conducted, dated 18.04.2024 and numbered 27,424. Informed consent and approval to participate were obtained from each participant. Permission was obtained for the use of the scale. It complies with the Helsinki Declaration. The clinical trial number was obtained on 18.09.2025 as NCT07179367.

### Statistical methods

The data obtained in the study were evaluated by using IBM SPSS Statistics for Windows, Version 22.0 (SPSS INC., Chicago, IL, USA) statistical software. Frequency and percentage analyses were used to determine the descriptive characteristics of the participants. Kurtosis and Skewness values were analysed to determine whether the research variables were normally distributed. Since the variables showed normal distribution, parametric methods were used in the analysis. Dependent groups t-test was used for the comparison of intragroup measurements. Independent groups t-test was used to compare quantitative continuous data between two independent groups. p-value of < 0.05 was interpreted as statistically significant.

## Results

According to participant information, the average age of the students was 23.5 years, with 69.6% being female. 84.8% graduated from Anatolian high schools, 8.7% from science high schools, and 6.5% from health vocational high schools. 43.5% had previously taken a patient safety course.

Time spent in each room was measured to evaluate the relative difficulty of the patient safety–related activities, with longer durations indicating increased complexity or student difficulty in completing the assigned tasks. It was observed that students spent the most time in the escape room with information questions, averaging 2.59 min. They spent an average of 1.18 min in the second room and 1.12 min in the third room. The average time for students to complete the escape room was 5.29 min (Table 3).

**Table 3:** Duration of stay in rooms

Rooms	Mean ± SD
First room	2.59 ± 0.85
Second room	1.18 ± 0.21
Third room	1.12 ± 0.32
Total time	5.29 ± 1.4

*SD* Standard Deviation

The mean score of the Patient Safety Self-Efficacy Questionnaire before the application was 61.26. The Cronbach’s Alpha value was found to be very high, at 0.977. After the application, the score was 71.32, showing an increase of 10.06 points. The Cronbach’s Alpha value was found to be very high, at 0.933. The increase in score after the application was significant (*p* <.05). Students reported high satisfaction with the escape room training method, with a satisfaction score of 73.71 (Table 4).

**Table 4:** Comparison of score averages from patient safety self-efficacy questionnaire before and after the escape room

Groups	Mean ± SD	t*	*p*
PSSE pre-test	61.26 ± 13.43	−5.176	< 0.000
PSSE post-test	71.32 ± 4.7		

*PSSE* Patient Safety Self-Efficacy, *n* Number of participants, *SD* Standard Deviation

*: Paired sample t-test

Fourth-year nursing students receive information on patient safety in all theoretical and practical courses they take throughout their undergraduate education. In addition, some students have taken elective courses specifically related to this topic. When analyzing the effect of taking a patient safety course on self-efficacy evaluations in the patient safety-themed escape room training method, it was found that whether or not students had taken the patient safety course did not create a significant difference in self-efficacy pre-test and post-test scores (*p* >.05). In intragroup comparisons, a significant increase of 12.05 points was observed in students who had taken the patient safety course (*p* <.05). Similarly, a significant increase of 8.54 points was observed in students who had not taken the patient safety course (*p* <.05) (Table 5).

**Table 5:** Comparison of score averages from patient safety self-efficacy test before and after the escape room according to patient safety course

Groups	Course taken (*n* = 20)	Course not taken (*n* = 26)	t^a^	*p*
	Mean ± SD	Mean ± SD		
PSSE pre-test	59.80 ± 16.96	62.38 ± 10.16	− 0.642	0.524
PSSE post-test	71.85 ± 3.26	70.92 ± 5.64	0.654	0.516
***t*** ^***b***^	−3.412	−4.01		
***p***	0.003	< 0.001		

*PSSE* Patient Safety Self-Efficacy, *n* Number of participants, *SD* Standard Deviation

^a^ T-Test in Independent Groups

^b^ T-Test in Dependent Groups

## Discussion

Patient safety requires the consistent application of critical thinking, problem-solving, and clinical decision-making skills [21]. Emerging evidence suggests that active teaching methods, including escape room pedagogy, can support the development of these core competencies in nursing education and thereby contribute to safer, higher-quality patient care [22, 23]. Accordingly, this study evaluated the impact of an escape room activity designed in accordance with the International Patient Safety Goals on nursing students’ self-efficacy regarding patient safety.

This study found that the escape room intervention was associated with a statistically significant improvement in patient safety self-efficacy, with mean self-efficacy scores increasing by 10.06 points (*p* <.05). By engaging students in realistic clinical scenarios within a controlled learning environment, the intervention may support safer decision-making and contribute to reducing the likelihood of patient harm in future clinical practice. In addition, students appeared to develop heightened awareness of patient safety principles. Consistent with our findings, previous studies have reported positive outcomes of patient safety–themed escape room activities, including increased confidence and meaningful shifts in learners’ perspectives among nursing and medical students [6, 11, 24].

Notably, the most challenging aspect for students was the task of sorting anatomical structures, with all groups spending the most time in the first room, where they answered questions related to patient safety and identified the ventrogluteal injection site. This finding aligns with the results of Molina-Torres et al. (2022), where students described the escape room as a game-based approach that offered a realistic and effective resource for anatomy learning [25].

According to the findings of this study, it can be said that it has a significant effect on increasing the self-efficacy of nursing students in patient safety practices. Based on this data, hypothesis H_1_^1^ is accepted.

Our study found that students reported high levels of satisfaction with the escape room training method, yielding a satisfaction score of 73.71. Similarly, previous research has observed that nursing students participating in escape room activities report higher information retention, increased motivation, and greater satisfaction [26–28].

Moreover, research has established that active learning approaches—including simulation, concept mapping, reflective learning, inverted classrooms, and escape rooms—are effective in developing critical thinking, communication, problem-solving, teamwork, and creative thinking skills [28–30]. Anguas-Gracia et al. (2021) found that students enjoyed game-based learning, were motivated to study more, and believed that such games should be more widely incorporated into nursing education [31]. They also reported that these games helped them retain theoretical knowledge and apply it in practice [30].

Feedback was collected during the debriefing phase using the Promoting Excellence and Reflective Learning in Simulation (PEARLS) method. In line with these findings, this research revealed that students valued the escape room method for its emphasis on teamwork and communication skills. They reported that the activity was enjoyable, informative, and memorable, with strong potential for practical application.

According to the findings of this study, it can be said that it has an effect on patient safety-themed escape room teaching method increases nursing students’ satisfaction. Based on this data, hypothesis H_1_^2^ is accepted.

## Conclusion

This study suggests that integrating patient safety–themed escape room training into the nursing curriculum can enhance students’ self-efficacy, satisfaction, and motivation. Based on these findings, nursing programs may consider incorporating escape room activities as an active learning strategy to support the development of key competencies, including critical thinking, teamwork, and communication. Furthermore, expanding patient safety–focused escape room training to interdisciplinary education and hospital in-service programs may strengthen patient safety culture and contribute to safer clinical practice.

### Limitations

A key limitation of this study is the use of a single-group pretest–posttest design, which limits causal inference regarding the educational effects of the intervention. Future studies employing controlled designs (e.g., randomized or quasi-experimental trials) are needed to more robustly evaluate the effectiveness of patient safety–themed escape room training.

## Supplementary Information

Supplementary Material 1.

## Data Availability

All data supporting the findings of this study are available within the paper and its Supplementary Information.
